# 日本の小学校算数と中学校数学における達成されたカリキュラム－全国学力・学習状況調査のアラインメント分析－

**DOI:** 10.12688/f1000research.175939.2

**Published:** 2026-07-09

**Authors:** 蒔苗直道 蒔苗

**Affiliations:** 1 Institute of Human Sciences, University of Tsukuba, Tsukuba, Ibaraki, 3058572, Japan

**Keywords:** Curriculum Alignment, Mathematics, National Assessment of Academic Ability

## Abstract

本稿では，日本の小学校算数および中学校数学における達成されたカリキュラムのアラインメントを全国学力・学習状況調査の結果から分析することを目的とする.このため，全国学力・学習状況調査のデータから，算数や数学の授業や指導の実施の程度に対する回答群間で，算数および数学の問題への平均正答率，領域ごと及び評価の観点ごとの平均正答率に差があるかを，一元配置分散分析により比較した.そして，この結果からアラインメントを分析すると，整合的なアラインメントの傾向が示唆された授業や指導の実施状況は，小学校算数における問題の解き方や考え方の過程が分かるように工夫してノートを書く指導，中学校数学における実生活における事象との関連を図った授業，観察や操作，実験等の活動を通して数量や図形等の性質を見いだす活動（ただし整合性は限定的），公式や決まりなどを指導するとき，生徒がその根拠を理解できるような工夫であった. なお，これらの関連は交絡要因を統制しない探索的な分析に基づくものであり，因果関係を示すものではない.

## Abstract 2

This study aims to analyze the alignment of the attained curriculum in Japanese elementary school arithmetic and junior high school mathematics, based on the results of the National Assessment of Academic Ability. To achieve this, data from the assessment were used to compare mean correct response rates across groups defined by the degree of implementation of each instructional practice, using one-way analysis of variance (ANOVA). These rates were considered both overall and by specific domains and evaluative perspectives. The analysis of alignment revealed several instructional practices that showed tendencies of alignment with attained learning outcomes. In elementary school arithmetic, alignment was observed in practices that encouraged students to write notes in ways that made the processes of problem solving and reasoning visible. In junior high school mathematics, alignment was evident in lessons that emphasized connections between mathematical concepts and real-life phenomena, as well as in instructional approaches that ensured students understood the rationale behind formulas and rules. These findings suggest that instructional practices explicitly fostering students’ comprehension of problem-solving processes, contextual relevance, and conceptual justification tend to show greater alignment with attained learning outcomes. However, as this study did not control for confounding variables, the observed associations should be interpreted as exploratory rather than causal. The study contributes to curriculum research by identifying associations between specific pedagogical practices and attained learning outcomes, thereby offering insights into effective instructional design in mathematics education.

## 1. はじめに

本稿の目的は，全国学力・学習状況調査の結果から，小学校算数および中学校数学における達成されたカリキュラムのアラインメントを分析することである.

達成されたカリキュラムとは，国際教育到達度評価学会 (IEA) が調査結果をまとめた際に示した 3 つのレベルのカリキュラム（意図されたカリキュラム (Intended Curriculum)，実施されたカリキュラム (Implemented Curriculum)，達成されたカリキュラム (Attained Curriculum)) の 1 つであり，実際に児童・生徒が学習した結果を意味している ( Travers et al., 1983). この3つのレベルのカリキュラムを用いて算数・数学の指導を捉えると，児童・生徒に獲得することが期待される学習内容が，実際に授業で教えられているか，また，児童・生徒に獲得されたのか，というアラインメント (Alignment) が教育上の課題となる ( Webb, 1997).

　「alignment」とは，「 （一列の）整列；一直線にすること」を第一義とし，「 （政治運動などの）連携，連合」や 「 （機械の部分品の）調整，整合」という日本語に訳される（ 小西・南出，2001，p.59）.日常では自動車の車軸調整を「アライメント調整」と呼んだり，ビジネス用語で部署や社員の最適化という意味で用いられたりする.日本の算数・数学教育の研究では，教育における目標・指導・評価が整合的かつ機能的に整列している状態として捉え，カタカナで「アラインメント」と表記され，近年のカリキュラム研究において着目されてきている（ 清水，2019， 2020）.

現行の学習指導要領への改訂がされて以降，日本のカリキュラムのアラインメントに関する研究では，意図されたカリキュラムと実施されたカリキュラムのアラインメントが取り上げられてきた. 例えば， 清水ら (2021) は中学校数学の「関数」領域に焦点を当てた教科書の分析を行い， 清水ら (2022) は小学校算数の「変化と関係」領域に焦点を当てた教科書の分析を行っている. 授業研究の文脈では， 清野ら (2022， 2023) は 2 つのカリキュラムの調整が行われ，教師がアラインメントの不整合を解消している実態を分析している. また ， 影山・斎藤 (2023) は多元分析アプローチを用いて，学習指導要領解説，学習指導案や板書計画，授業中の発話，板書，授業後インタビューの発話のアラインメントを検証している. こうした研究では，達成されたカリキュラムの分析が行われておらず，一連のアラインメント研究において残された課題となっている.

　達成されたカリキュラムを取り上げる方法としては，全国学力・学習状況調査の利用が指摘されているが（ 笠井・水谷，2021； 清水・宮崎，2024），実際のアラインメント分析は行われていない.こうした研究の状況から，本稿では，全国学力・学習状況調査の結果 ^1^ から，小学校算数および中学校数学における達成されたカリキュラムのアラインメントを分析することを目的とする.

## 2.アラインメントを捉える理論枠組み

### (1) 達成されたカリキュラムのアラインメント分析の焦点

　3 つのレベルのカリキュラム間のアラインメントは， 宮崎 (2019) によって 図 1 のようなモデルで説明されている.この図の A1 は，意図されたカリキュラムから実施されたカリキュラム ^2^ が形成される作用，A2 は，実施されたカリキュラムから意図されたカリキュラムが形成される作用を表している. 同様に，B1 は，実施されたカリキュラムから達成されたカリキュラム，B2 は，達成されたカリキュラムから実施されたカリキュラム，C1 は，達成されたカリキュラムから意図されたカリキュラム，C2 は，意図されたカリキュラムから達成されたカリキュラムへの形成作用と説明され，アラインメントには双方向性があることが指摘されている.

**図1.**
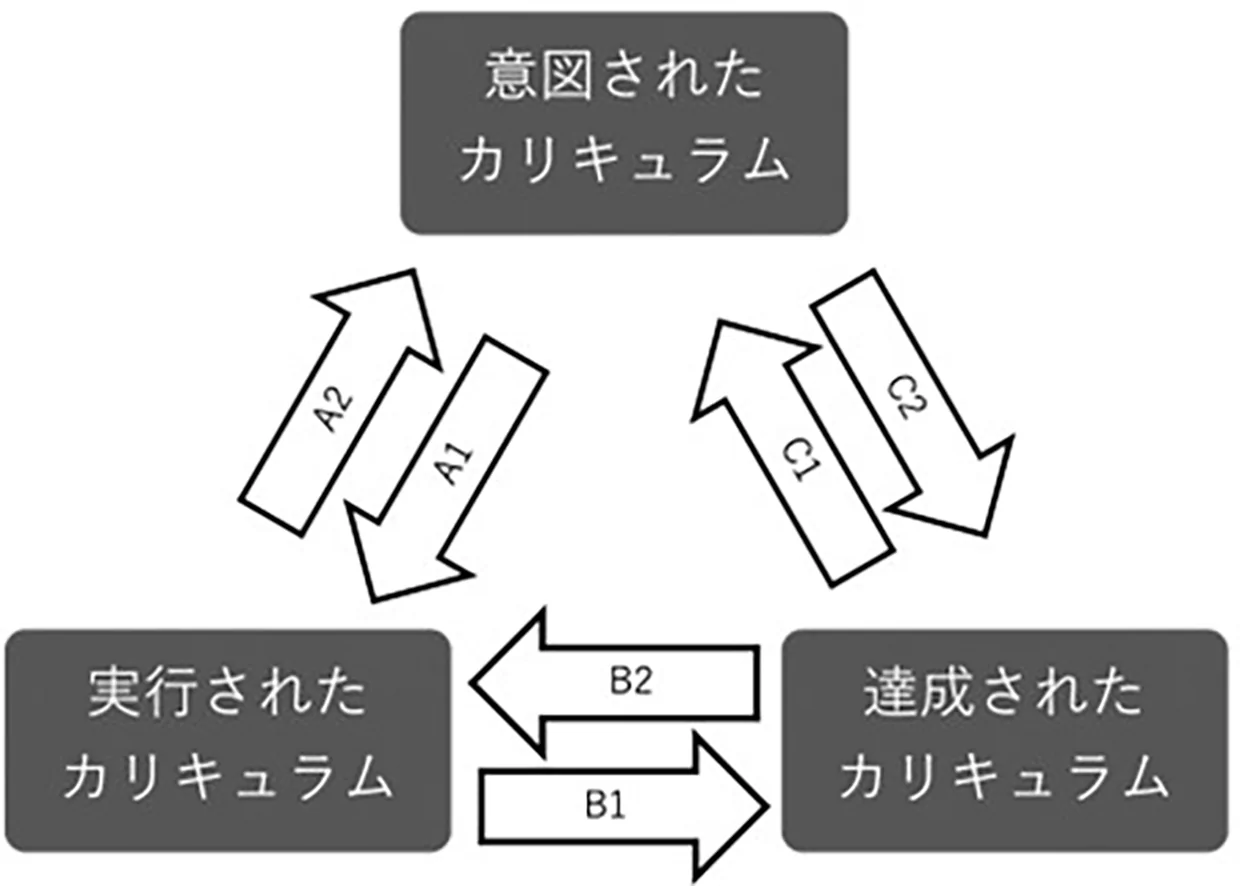
3つのカリキュラム間のアラインメント. （ 宮崎，2019，p.102 より作成　）.

　そして，教育組織を教育を統轄する仕組みとして捉える際には，国や地方自治体などの行政レベルの層と，学校や学級などの実際に教育実践に当たる実践レベルの層との 2 層に分けて捉える事ができるとされている. この考えを反映して修正された枠組みが， 図2 の「 【二層―双方向】型アラインメント」である.

　この枠組みに対応する行政レベルでの意図されたカリキュラムには学習指導要領が示され，実施されたカリキュラムには指定事業等の授業，達成されたカリキュラムには全国学力・学習状況調査が例示されている.本稿における達成されたカリキュラムのアラインメント分析は， 図1 では B1 と B2 の実施されたカリキュラムと達成されたカリキュラムのアラインメントに該当し， 図2 では層アである行政レベルにおけるアラインメントに該当する.

**図2.**
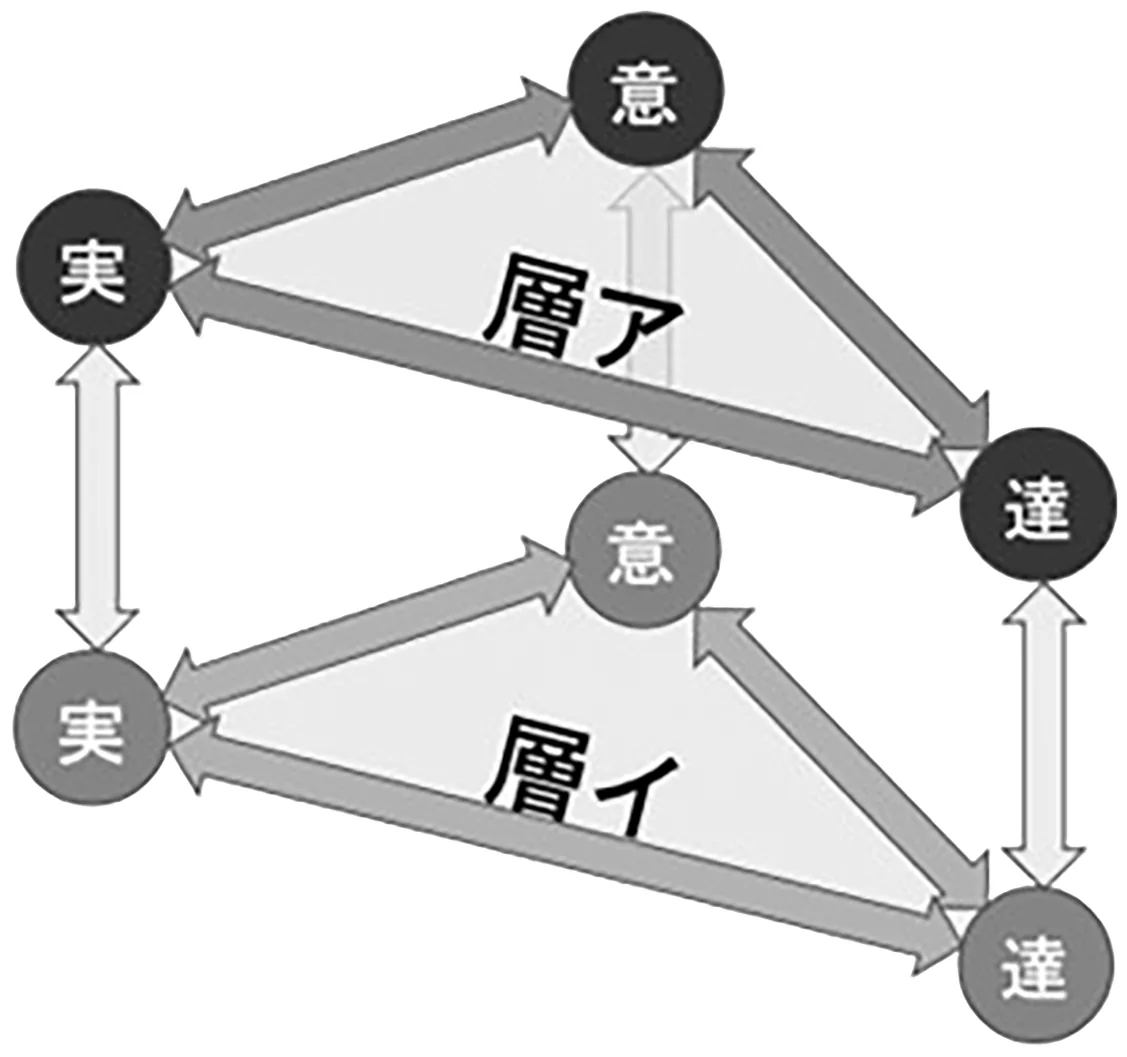
【二層―双方向】型アラインメント. （ 宮崎，2021，p.236 より作成　）.

　ただし，指定事業等の授業を実施されたカリキュラムとし，全国学力・学習状況調査を達成されたカリキュラムとすると，そのアラインメントの分析では，特定の授業と全国的な動向を比較することになり，分析対象の次元が異なってしまう. 統括する組織レベルを統一するのみではなく，全国的な動向として実施されたカリキュラムを捉え直し，分析対象の次元を統一する必要がある.

### (2) 実施されたカリキュラムとしての算数および数学の指導

　実施されたカリキュラムは実際の算数および数学の授業で教えられた内容を意味しているが，アラインメントの分析に当たっては，その取り上げ方や指導方法にも着目する必要がある.全国学力・学習状況調査の数学の問題は，学習指導要領に示されている指導内容を前提に出題されており，この意味では，学習指導要領に示された指導は，全国の学校で実施されているという前提で調査されている.このため，調査の報告書にまとめられた調査結果の概要や各問題の正答率などは，達成されたカリキュラムと解釈することができる.

　その反面，調査の報告書には，調査結果の正答率から学習指導の改善に向けた提言や出題された問題を用いた授業アイディア例が示されている.これは，実際の授業における指導には課題があることを指摘しており，実施されたカリキュラムは学習指導要領に示されたとおりに実施されているわけではないことを表している.この意味で，全国学力・学習状況調査の報告書には，指導内容が全国の学校で実施されているという前提とその実施上に課題があるという指摘との両面が含まれている.

　このように実施されたカリキュラムを学習指導要領の指導内容のみから捉えるには問題点が存在するわけであるが，別の観点から捉える必要性を指摘することもできる。学習指導要領では，数学的活動を通して資質・能力を育成することが，算数科と数学科の目標とされ，指導内容においても数学的活動の項目が示されている. 単に，算数または数学の指導内容を取り上げるのでは，実施されたカリキュラムの捉えは不十分であり，数学的な活動を通した指導が実際の実施されたカリキュラムに伴うことを踏まえなければならない. つまり，学習指導要領の指導内容については学校で指導されているという前提の下で，指導内容の取り上げ方や指導方法にも着目することが，実施されたカリキュラムを捉える上で必要なのである.

　この点について，全国学力・学習状況調査の学校質問紙では小学校算数について「算数科の指導方法」（ 文部科学省・国立教育政策研究所，2021a，pp.42-44），中学校数学について「数学科の指導方法」（ 文部科学省・国立教育政策研究所，2022a，p.29）についての質問項目がある. 学校質問紙は，調査に参加した児童や生徒の所属する学校が回答するものであり，算数や数学の指導の実施状況の全国的な動向を調査したものである.これは，学校長の責任において回答する事になっており，質問にできるだけ正確に答えるため，必要に応じて関係する教職員に状況を確認して回答する事が質問紙の冒頭に記されている. 本稿では実施されたカリキュラムの指導の実質として，学校質問紙における「算数科の指導方法」と「数学科の指導方法」に取り上げられた内容から全国な動向としての実施されたカリキュラムを捉えるものとする.

　なお，本研究で用いる匿名データは個票単位で貸与されており，各児童生徒の記録には所属校が学校質問紙に回答した値が割り当てられている. 本稿の分析は，この個票データを単位として実施しているため，集計表に示される度数は児童生徒数を表す. 同一校に所属する児童生徒は同じ学校質問紙回答を共有するため，本分析における観察値は厳密な意味で独立ではない. この点については，本研究の限界として「5. おわりに (2) 本研究の限界と今後の課題」で改めて論じる.

## 3.アラインメントの分析

### (1) 小学校算数

　小学校算数のアラインメント分析に用いる全国学力・学習状況調査のデータは，現行の学習指導要領が全面実施された令和 2 年度の指導を反映した令和 3 年 5 月 27 日実施の令和 3 年度の全国学力・学習状況調査のデータを用いる. データは，文部科学省より貸与を受けた匿名データで，小学校に在籍し，5 月 27 日に調査を受けた第 6 学年の児童から約 10% をランダム抽出した 99,445 人分である. 全国学力・学習状況調査は悉皆調査であり，令和 3 年度調査の小学校第 6 学年の集計数は 1,005,600 人である（ 文部科学省・国立教育政策研究所，2021b，p4）. 匿名データは，この集計数に対して 10% 未満の集計数であるが，全国的な傾向を把握する上では，十分なデータである.

①　変数

(i)　実施されたカリキュラム

　実施されたカリキュラムとして取り上げる令和 3 年度の全国学力・学習状況調査における学校質問紙では，「新型コロナウィルス感染症の影響」についての質問項目 (I ~ XI) を除くと，この年の質問項目は (1) ~ (91) であり，そのうち，算数科の指導方法として，以下に示す質問 (55) から (60) の 6 つの質問がされている. 回答は4件法 (1. よく行った，2. どちらかといえば，よく行った，3. あまり行わなかった，4. 全く行わなかった）で収集されている. このうち，算数科の授業について具体的な指導方法を尋ねている質問の番号 (57) (58) (59) (60) を分析対象とする.

「調査対象である第 6 学年の児童に対する算数の授業において，前年度までに，次のような指導をどの程度行いましたか. 当てはまる番号を1つずつ選んでください.

(55) 補充的な学習の指導

(56) 発展的な学習の指導

(57) 実生活における事象との関連を図った授業

(58) 具体的な物を操作するなどの体験を伴う学習を通して，数量や図形について実感を伴った理解をする活動

(59) 公式やきまり，計算の仕方等を指導するとき，児童がそのわけを理解できるように工夫していた

(60) 問題の解き方や考え方の過程が分かるように工夫してノートを書く指導」

（ 文部科学省・国立教育政策研究所，2021a，pp.42-44）

　なお，分析対象とした 99,445 人のうち，学校質問紙の各質問項目について無回答・欠測のある児童が存在した. 各質問の有効回答数は，質問 (57) が 99,159 人 (99.7%)，質問 (58) が 99,253 人 (99.8%)，質問 (59) が 99,209 人 (99.8%)，質問 (60) が 99,197 人 (99.7%) であった. 各分析では，当該質問項目に有効回答のあったケースのみを対象とした（リストワイズ除外）. 有効回答率はいずれも 99.7% を上回っており，欠測による偏りは限定的と考えられる.

(ii) 　達成されたカリキュラム

　達成されたカリキュラムを捉えるために取り上げる算数の問題は，16 問出題され，その集計結果は 表1 のように報告されている. また，問題別集計結果は， 表2 のようにまとめられる. なお，正答率と無解答率には，本研究で用いる匿名データの集計結果を追記している. 調査全体の集計結果と本研究で用いる匿名データの結果には，大きな違いは認められない.

**表1.** 算数の集計結果.

児童数	1,005,600人
	99,445人
平均正答数	11.3問
	11.2問
平均正答率	70.3%
	70.2%
中央値	12.0問
	12.0問
標準偏差	3.5問
	3.5問
最頻値	14問
	14問

（ 文部科学省・国立教育政策研究所，2021b，p.10）より作成　下段は匿名データの集計結果.

**表2.** 小学校算数の問題別集計結果.

問題番号	問題の概要	学習指導要領の領域					評価の観点			問題形式			正答率(%)	無解答率(%)
		数と計算	図形	測定	変化と関係	データの活用	知識･技能	思考･判断･表現	主体的に学習に取り組む態度	選択式	短答式	記述式		
1(1)	二つのコースの道のりの求め方と答えを書く	○		○				○				○	62.7 62.4	1.7 1.7
1(2)	500m を歩くのに 7 分間かかることを基に，1000m を歩くのみかかる時間を書く				○			○			○		86.8 86.7	1.7 1.7
1(3)	アとイの二つの速さを求める式の意味について，正しいものを選ぶ				○		○			○			56.0 56.0	1.4 1.4
1(4)	午前 1 時 35 分から 50 分後の時刻を書く			○			○				○		89.3 89.4	0.7 0.6
1(5)	分速 540m のバスが 2700m 進むのにかかる時間を求める式を書く				○		○				○		85.2 85.1	1.5 1.5
2(1)	直角三角形の面積を求める式を書く		○				○				○		55.4 55.3	1.5 1.5
2(2)	直角三角形を組み合わせた図形の面積について分かることを書く		○	○			○			○			72.7 72.4	1.0 1.0
2(3)	二等辺三角形を組み合わせた平行四辺形の面積の求め方と答えを書く		○					○				○	46.2 46.1	4.5 4.5
3(1)	6 年生の本の貸し出し冊数を，棒グラフから読み取って選ぶ					○	○			○			95.8 95.9	0.4 0.3
3(2)	学年ごとの本の貸し出し冊数について，棒グラフから分かることを選ぶ					○	○			○			90.8 90.7	0.4 0.4
3(3)	「114」は二次元の表のどこに入るかを選ぶ					○	○			○			67.7 67.4	1.7 1.8
3(4)	帯グラフから，割合の違いが，一番大きい項目を選び，その項目と割合を書く					○		○				○	52.2 51.9	10.3 10.1
3(5)	5 年生と 6 年生の読みたい本と，大きくの 5 年生と 6 年生に読まれている本を調べるために，適切なデータを選ぶ.					○		○		○			74.1 73.9	1.3 1.3
4(1)	余りのある除法の商と余りを基に，23個のボールを6個ずつ箱にいれていくときに必要な箱の数を書く	○						○			○		83.1 83.1	1.7 1.7
4(2)	8 人に4Lのジュースを等しく分けるときの一人分のジュースの量を求める式と答えを書く	○					○				○		55.7 55.7	2.0 1.9
4(3)	30m を 1 としたときに，12m が 0.4 に当たるわけを書く	○						○				○	51.6 51.4	10.3 10.3

　領域ごとの出題は，数と計算が4問，図形が 3 問，測定が 3 問，変化と関係が 3 問，データの活用が5問となっている.このうち，複数の領域に関する問題は，数と計算と測定が1問，図形と測定が 1 問出題されている. 評価の観点では，知識・理解が 9 問，思考・判断・表現が7問，主体的に学習に取り組む態度については出題がなかった.

② 手続き

(i)　算数の正答率とのアラインメント

　達成されたカリキュラムのアラインメントの分析では，第一に，学校質問紙の 4 つの授業や指導の実施の程度に対する回答群間で，算数の正答率の平均に差があるかを，一元配置分散分析（有意水準 5%）により検討する. これにより，算数の達成度に対する実施された授業や指導とのアラインメントを分析する.

(ii)　領域ごとの正答率とのアラインメント

　第二に，各領域の正答率に，学校質問紙の 4 つの授業や指導の実施の程度に対する回答群間で，各領域の正答率の平均に差があるかを，一元配置分散分析（有意水準 5%）により検討する. これにより，算数の各領域の達成度に対する実施された授業や指導とのアラインメントを分析する.

(iii)　評価の観点ごとの正答率とのアラインメント

　第三に，評価の観点ごとの正答率に，学校質問紙の 4 つの授業や指導の実施の程度に対する回答群間で，評価の観点ごとの正答率の平均に差があるかを，一元配置分散分析（有意水準 5%）により検討する.これにより，評価の観点ごとの算数の達成度に対する実施された授業や指導とのアラインメントを分析する.

③ 分析結果

(i)　算数の正答率とのアラインメント

表3 は，学校質問紙の質問 (57) (58) (59) (60) の回答と算数の正答率の平均を集計したものである. 質問 (57) (58) (60) に対して，肯定的 ^3^ に回答している方が，算数の正答率が高くなっている. 質問 (59) に対しては，4 の正答率が最も高く，その次に 1，2，3 の順になっている.

**表3.** 学校質問紙の質問 (57) (58) (59) (60) の回答と算数の正答率の平均.

	回答			
	1	2	3	4
質問 (57)	70.6%	70.2%	69.7%	69.6%
	16553	64497	17955	154
質問 (58)	70.5%	70.1%	70.1%	69.1%
	29352	61845	8012	44
質問 (59)	70.1%	69.7%	67.9%	71.5%
	42823	53966	2386	34
質問 (60)	71.0%	69.9%	68.4%	63.5%
	39791	53415	5953	38

下段は度数.

表4 は，質問 (57) (58) (60) について，分散分析を行った結果である.学校質問紙の質問 (57) (60) について， 5% の有意水準で有意差が認められる. 質問 (58) については，有意差は認められない.

**表4.** 学校質問紙の質問 (57) (58) (60) の回答と算数の正答率の平均の分散分析.

	平方和	自由度	平均平方	F 値	有意確率
質問(57)	8021.321	3	2673.774	5.627	<.001 ^†^
質問(58)	3304.697	3	1101.566	2.319	.073
質問(60)	50050.181	3	16683.394	35.143	<.001 ^†^

^†^ *p* < .05.

(ii)　領域ごとの達成度に対する実施された授業や指導のアラインメント

A　実生活における事象との関連を図った授業

表5 は，学校質問紙の質問 (57) の回答と各領域の正答率の平均を集計したものである. 数と計算，図形，測定の 3 つの領域においては，肯定的に回答している方が，正答率が高くなっている. 変化と関係とデータの活用の 2 つの領域は 1 の正答率が最も高く，その次に 4，2，3 の順になっているが，最も高い正答率と最も低い正答率の差が 1% 未満である.

**表5.** 学校質問紙の質問 (57) の回答と各領域の正答率の平均.

	回答			
	1	2	3	4
数と計算	63.9%	63.2%	62.6%	60.9%
図形	58.3%	58.0%	57.3%	57.1%
測定	75.3%	74.7%	74.3%	72.3%
変化と関係	76.1%	75.9%	75.6%	76.0%
データの活用	76.5%	76.0%	75.4%	76.2%

表6 は，数と計算，図形，測定の 3 つの領域の結果について，分散分析を行った結果である. 数と計算，図形，測定 3 つの領域において，5% の有意水準で有意差が認められる.

**表6.** 学校質問紙の質問 (57) の回答と各領域の正答率の平均の分散分析.

	平方和	自由度	平均平方	F 値	有意確率
数と計算	14600.098	3	4866.699	5.100	.002 ^†^
図形	10853.498	3	3617.833	3.187	.023 ^†^
測定	9499.407	3	3166.469	4.295	.005 ^†^

^†^ *p* < .05.

B　具体的な物を操作するなどの体験を伴う学習を通して，数量や図形について実感を伴った理解をする活動

表7 は，学校質問紙の質問 (58) の回答と各領域の正答率の平均を集計したものである. 数と計算の領域と変化と関係の領域においては，肯定的に回答している方が，正答率の平均が高くなっている. 図形の領域においては，4 に回答した正答率の平均が最も高く，その次に 1，3，2 の順になっている. 測定の領域においては，1 に回答した正答率の平均が最も高く，その次に 4，2，3 の順になっている. データの活用の領域においては，4 に回答した正答率の平均が最も高く，その次に 1，2，3 の順になっている.

**表7.** 学校質問紙の質問 (58) の回答と各領域の正答率の平均.

	回答			
	1	2	3	4
数と計算	63.5%	63.0%	62.9%	56.8%
図形	58.2%	57.8%	58.1%	59.8%
測定	75.1%	74.6%	74.5%	75.0%
変化と関係	76.1%	75.7%	76.0%	72.7%
データの活用	76.2%	75.9%	75.7%	77.7%

表8 は，数と計算の領域と変化と関係の領域の結果について，分散分析を行った結果である.数と計算の領域については，5% の有意水準で有意差が認められる.変化と関係の領域については，有意差は認められない.

**表8.** 学校質問紙の質問 (58) の回答と各領域の正答率の平均の分散分析.

	平方和	自由度	平均平方	F 値	有意確率
数と計算	7579.82	3	2526.608	2.648	.047 ^†^
変化と関係	2971.642	3	990.547	1.333	.261

^†^ *p* < .05.

C　公式やきまり，計算の仕方等を指導するとき，児童がそのわけを理解できるような工夫

表9 は，学校質問紙の質問 (59) の回答と各領域の正答率の平均を集計したものである.数と計算の領域と測定の領域とデータの活用の 3 つの領域においては，1 に回答した正答率の平均が最も高く，その次に4，2，3 の順になっている. 図形の領域と変化と関係の 2 つの領域においては，4 に回答した正答率の平均が最も高く，その次に 1，2，3 の順になっている. 肯定的に回答している方が，正答率の平均が高くなるような領域はない.

D　問題の解き方や考え方の過程が分かるように工夫してノートを書く指導

**表9.** 学校質問紙の質問 (59) の回答と各領域の正答率の平均.

	回答			
	1	2	3	4
数と計算	64.1%	62.5%	60.7%	62.5%
図形	59.0%	57.2%	55.3%	60.8%
測定	75.3%	74.3%	73.1%	74.5%
変化と関係	76.4%	75.5%	74.3%	79.4%
データの活用	76.7%	75.6%	73.0%	75.9%

表10 は，学校質問紙の質問 (60) の回答と各領域の正答率の平均を集計したものである. 数と計算，図形，測定.変化と関係，データの活用のすべての領域において，肯定的に回答している方が，正答率が高くなっている.

**表10.** 学校質問紙の質問 (60) の回答と各領域の正答率の平均.

	回答			
	1	2	3	4
数と計算	64.0%	62.8%	61.0%	54.6%
図形	58.8%	57.6%	55.8%	50.9%
測定	75.3%	74.5%	72.9%	64.0%
変化と関係	76.5%	75.6%	74.6%	67.5%
データの活用	76.7%	75.6%	74.1%	72.1%

表11 は，上記の結果について，分散分析を行った結果である.数と計算，図形，測定，変化と関係，データの活用のすべての領域において，5% の有意水準で有意差が認められる.

**表11.** 学校質問紙の質問 (60) の回答と各領域の正答率の平均の分散分析.

	平方和	自由度	平均平方	F 値	有意確率
数と計算	69369.910	3	23123.303	24.252	<.001 ^†^
図形	63472.163	3	21157.388	18.644	<.001 ^†^
測定	41056.973	3	13685.658	18.570	<.001 ^†^
変化と関係	33807.318	3	11269.106	15.176	<.001 ^†^
データの活用	50824.074	3	16941.358	29.404	<.001 ^†^

^†^ *p* < .05.

(iii)　評価の観点ごとの達成度に対する実施された授業や指導のアラインメント

A　実生活における事象との関連を図った授業

表12 は，学校質問紙の質問 (57) の回答とその評価の観点ごとの正答率の平均を集計したものである. 知識・技能の評価の観点においては，肯定的に回答している方が，正答率が高くなっている.思考・判断・表現の評価の観点においては，1 に回答した正答率の平均が最も高く，その次に 2，4，3 の順になっている.

**表12.** 学校質問紙の質問 (57) の回答と評価の観点別の正答率の平均.

	回答			
	1	2	3	4
知識・技能	74.4%	74.3%	73.7%	73.4%
思考・判断・表現	65.8%	65.1%	64.5%	64.7%

表13 は，知識・技能の結果について，分散分析を行った結果である. 5% の有意水準で有意差が認められる.

**表13.** 学校質問紙の質問 (57) の回答と評価の観点別の正答率の平均の分散分析.

	平方和	自由度	平均平方	F 値	有意確率
知識・技能	4493.537	3	1497.846	3.371	.018 ^†^

^†^ *p* < .05

B　具体的な物を操作するなどの体験を伴う学習を通して，数量や図形について実感を伴った理解をする活動

表14 は，学校質問紙の質問 (58) の回答とその評価の観点ごとの正答率の平均を集計したものである.知識・技能の評価の観点においては，4 に回答した正答率の平均が最も高く，その次に 1，2，3 の順になっている. 思考・判断・表現の評価の観点においては，1 に回答した正答率の平均が最も高く，その次に 3，2，4 の順になっている.

**表14.** 学校質問紙の質問 (58) の回答と評価の観点別の正答率の平均.

	回答			
	1	2	3	4
知識・技能	74.4%	74.1%	74.1%	75.3%
思考・判断・表現	65.5%	64.9%	65.1%	61.4%

C　公式やきまり，計算の仕方等を指導するとき，児童がそのわけを理解できるような工夫

表15 は，学校質問紙の質問 (59) の回答とその評価の観点ごとの正答率の平均を集計したものである. 知識・技能，思考・判断・表現の両方の評価の観点において，4 に回答した正答率の平均が最も高く，その次に 1，2，3 の順になっている.

**表15.** 学校質問紙の質問 (59) の回答と評価の観点別の正答率の平均.

	回答			
	1	2	3	4
知識・技能	74.8%	73.8%	72.2%	75.5%
思考・判断・表現	66.0%	64.5%	62.4%	66.4%

D　問題の解き方や考え方の過程が分かるように工夫してノートを書く指導

表16 は，学校質問紙の質問 (60) の回答とその評価の観点ごとの正答率の平均を集計したものである. 知識・技能，思考・判断・表現の評価の両方の観点においては，肯定的に回答している方が，正答率が高くなっている.

**表16.** 学校質問紙の質問 (60) の回答と評価の観点別の正答率の平均.

	回答			
	1	2	3	4
知識・技能	74.8%	73.9%	72.6%	70.8%
思考・判断・表現	66.0%	64.6%	63.1%	54.1%

表17 は，上記の結果について，分散分析を行った結果である. 知識・技能，思考・判断・表現の両方の評価の観点において，5% の有意水準で有意差が認められる.

**表17.** 学校質問紙の質問 (60) の回答と評価の観点別の正答率の平均の分散分析.

	平方和	自由度	平均平方	F 値	有意確率
知識・技能	33531.973	3	11177.324	25.172	<.001 ^†^
思考・判断・表現	77758.111	3	25919.370	34.984	<.001 ^†^

^†^ *p* < .05.

(2) 中学校数学

　中学校数学のアラインメント分析に用いる全国学力・学習状況調査のデータは，現行の学習指導要領が全面実施された令和 3 年度の指導が反映された令和 4 年 4 月 19 日実施の令和 4 年度調査を対象とする. データは，文部科学省から貸与された匿名データで，中学校に在籍し，4 月 19 日に調査を受けた第 3 学年の生徒から約 10% をランダム抽出した 89,435 人分のデータである. 全国学力・学習状況調査は悉皆調査であり，令和4年度調査の中学校第 3 学年の集計数は 928,509 人である（ 文部科学省・国立教育政策研究所，2022b，p.4）. 匿名データは，この集計数に対して 10% 未満の集計数であるが，全国的な傾向を把握する上では，十分なデータである.

①　変数

(i)　実施されたカリキュラム

　実施されたカリキュラムとして取り上げる令和 4 年度の全国学力・学習状況調査における学校質問紙では，「新型コロナウィルス感染症の影響」についての質問項目 (I~III) を除くと，質問項目は (1)~(80) であり，そのうち，数学科の指導方法については (46) (47) (48) の 3 つの質問がされている. 回答は 4 件法 （1. よく行った，2. どちらかといえば，よく行った，3. あまり行わなかった，4. 全く行わなかった）で収集されている. この 3 つの質問項目を分析対象とする.

「調査対象である第 3 学年の生徒に対する数学の授業において，前年度までに，次のような指導をどの程度行いましたか.当てはまる番号を 1 つずつ選んでください.

(46) 実生活における事象との関連を図った授業

(47) 観察や操作，実験等の活動を通して，数量や図形等の性質を見いだす活動

(48) 公式や決まりなどを指導するとき，生徒がその根拠を理解できるように工夫していた」

（ 文部科学省・国立教育政策研究所，2022a，p.29）

なお，分析対象とした 89,435 人のうち，学校質問紙の数学科指導方法に関する 3 つの質問項目すべてに有効な回答が得られた 89,170 人（有効回答率 99.7%）を各分析の対象とした.

(ii)　達成されたカリキュラム

　達成されたカリキュラムを捉えるために取り上げる数学の問題は 14 問出題され，その集計結果は 表18 のように報告されている. また，問題別集計結果は， 表19ようにまとめられる. なお，正答率と無解答率には，本研究で用いる匿名データの数値を追記している. 調査全体の集計結果と本研究で用いる匿名データの結果には，大きな違いは認められない.

**表18.** 数学の集計結果.

平均正答数	7.3問
	7.2問
平均正答率	52.0%
	51.4%
中央値	8.0問
	7.0問
標準偏差	3.6問
	3.6問
最頻値	9問
	9問

（ 文部科学省・国立教育政策研究所，2022b，p.10）より作成　下段は匿名データの集計結果.

**表19.** 中学校数学の問題別集計結果.

問題番号	問題の概要	学習指導要領の領域				評価の観点			問題形式			正答率(%)	無解答率(%)
		数と式	図形	関数	データの活用	知識･技能	思考･判断･表現	主体的に学習に取り組む態度	選択式	短答式	記述式		
1	42 を素因数分解する	○				○				○		52.9 52.0	11.3 11.6
2	連立二元一次方程式 $\left\{ \begin{array}{c} 2x+y=1\\ y=x+4 \end{array}\right.$ を解く	○				○				○		75.1 74.5	5.8 6.1
3	ある予想がいつも成り立つかどうかを示すことについて，正しく述べたものを選ぶ		○			○			○			45.5 45.0	0.4 0.4
4	変化の割合が 2 である一次関数の関係を表した表を選ぶ			○		○			○			38.7 37.7	0.4 0.4
5	容器のふたを投げたときに下向きになる確率を選ぶ				○	○			○			83.6 83.4	0.3 0.3
6(1)	同じ偶数の和である 2n+2n=4N について，n が 9 のときどのような計算を表しているかを書く	○				○				○		74.4 73.9	5.8 5.9
6(2)	差が 4 である偶数の和が，4 の倍数になることの説明を完成する	○					○				○	49.5 48.9	19.6 20.0
6(3)	ある偶数との和が 4 の倍数になる数について，予想した事柄を表現する	○					○				○	38.2 37.6	25.8 26.3
7(1)	コマ回し大会で使用するコマをヒストグラムの特徴を基に選び，選んだ理由を説明する				○		○				○	44.2 44.1	0.7 1.3
7(2)	箱ひげ図の箱が示す区間に含まれているデータの個数と散らばりの程度について，正しく述べたものを選ぶ				○	○			○			44.4 44.2	0.7 0.7
8(1)	与えられたグラフにおいて，点Eの座標を書く			○		○				○		55.3 54.3	7.0 7.2
8(2)	目標の 300kg を達成するまでの日数を求める方法を説明する			○			○				○	39.0 38.3	24.0 24.6
9(1)	証明で用いられている三角形の合同条件を書く		○			○				○		73.6 73.3	7.3 7.5
9(2)	∠ABE と ∠CBF の和が 30° になる理由を示し，∠EBF の大きさがいつでも 60° になることの説明を完成 する		○				○			○		13.3 12.6	38.0 38.3

　領域ごとの出題は，数と式が 5 問，図形が 3 問，関数が 3 問，データの活用が 3 問となっている. 評価の観点では，知識・理解が9問，思考・判断・表現が 5 問，主体的に学習に取り組む態度については出題がなかった.

② 手続き

(i)　数学の正答率とのアラインメント

　達成されたカリキュラムのアラインメントの分析では，第一に，学校質問紙の 3 つの授業や指導の実施の程度に対する回答群間で，数学の正答率の平均に差があるかを，一元配置分散分析（有意水準 5%）により検討する.これにより，数学の達成度全般に対する実施された授業や指導とのアラインメントを分析 する.

(ii)　領域ごとの正答率とのアラインメント

　第二に，各領域の正答率に，学校質問紙の 3 つの授業や指導の実施の程度に対する回答群間で，各領域の正答率の平均に差があるかを，一元配置分散分析（有意水準 5%）により検討する.これにより，各領域の数学の達成度に対する実施された授業や指導とのアラインメントを分析する.

(iii)　評価の観点ごとの正答率とのアラインメント

　第三に，評価の観点ごとの正答率に，学校質問紙の3つの授業や指導の実施の程度に対する回答群間で，評価の観点ごとの正答率の平均に差があるかを，一元配置分散分析（有意水準 5%）により検討する.これにより，評価の観点ごとの数学の達成度に対する実施された授業や指導とのアラインメントを分析する.

③ 分析結果

(i)　数学の正答率とのアラインメント

表20 は，学校質問紙の質問 (46) (47) (48) の回答に対する数学の正答率の平均を集計したものである. すべての質問に対して，肯定的に回答している方が，数学の正答率が高くなっている. なお，質問 (48) について，4 と回答したものは含まれていなかったため，正答率は求められていない.

**表20.** 学校質問紙の質問 (46) (47) (48) の回答と数学の正答率の平均.

	回答			
	1	2	3	4
質問 (46)	52.6%	51.4%	50.4%	47.8%
	14662	58681	15637	190
質問 (47)	52.9%	51.4%	50.5%	50.3%
	14749	54340	19555	526
質問 (48)	52.3%	50.9%	49.1%	-
	38154	48715	2301	0

下段は度数.

表21 は，上記の結果について，分散分析を行った結果である. すべての質問について，5% の有意水準で有意差が認められる.

**表21.** 学校質問紙の質問 (46) (47) (48) の回答と数学の正答率の平均の分散分析.

	平方和	自由度	平均平方	F 値	有意確率
質問 (46)	40173.527	3	13391.176	20.186	<.001 ^†^
質問 (47)	53490.828	3	17830.276	26.884	<.001 ^†^
質問 (48)	56362.737	2	28181.369	42.494	<.001 ^†^

^†^ *p* < .05.

(ii)　領域ごとの正答率のアラインメント

A 実生活における事象との関連を図った授業

表22 は，学校質問紙の質問 (46) の回答に対する各領域の正答率の平均を集計したものである. すべての領域において，肯定的に回答している方が，正答率が高くなっている.

**表22.** 学校質問紙の質問 (46) の回答と各領域の正答率の平均.

	回答			
	1	2	3	4
数と式	58.7%	57.3%	56.3%	52.5%
図形	44.9%	43.6%	42.8%	41.4%
関数	44.7%	43.5%	42.1%	38.8%
データの活用	58.0%	57.3%	56.4%	55.3%

表23 は，上記の結果について，分散分析を行った結果である.すべての領域において，5% の有意水準で有意差が認められる.

**表23.** 学校質問紙の質問 (46) の回答と各領域の正答率の平均の分散分析.

	平方和	自由度	平均平方	F 値	有意確率
数と式	49341.536	3	16447.179	14.722	<.001 ^†^
図形	38284.955	3	12761.655	14.962	<.001 ^†^
関数	55818.104	3	18606.035	15.001	<.001 ^†^
データの活用	19936.437	3	6645.479	7.503	<.001 ^†^

^†^ *p* < .05.

B 観察や操作，実験等の活動を通して，数量や図形等の性質を見いだす活動

表24 は，学校質問紙の質問 (47) の回答に対する各領域の正答率の平均を集計したものである. 数と式とデータの活用の 2 つの領域は，肯定的に回答している方が，正答率が高くなっている. 図形の領域は，1 と回答した正答率が最も高く，その次に 4，2，3 の順になっている. 関数の領域は，1 と回答した正答率が最も高く，その次に 2，4，3 の順になっている.

**表24.** 学校質問紙の質問 (47) の回答と各領域の正答率の平均.

	回答			
	1	2	3	4
数と式	59.2%	57.3%	56.3%	56.1%
図形	45.2%	43.5%	42.8%	44.6%
関数	45.1%	43.4%	42.3%	42.8%
データの活用	58.1%	57.3%	56.4%	53.9%

表25 は，数と式の領域とデータの活用の領域の結果について，分散分析を行った結果である. 両方の領域において，5% の有意水準で有意差が認められる.

**表25.** 学校質問紙の質問 (47) の回答と各領域の正答率の平均の分散分析.

	平方和	自由度	平均平方	F 値	有意確率
数と式	69518.236	3	23172.745	20.747	<.001 ^†^
データの活用	30768.327	3	10256.109	11.582	<.001 ^†^

^†^ *p* < .05.

C　公式や決まりなどを指導するとき，生徒がその根拠を理解できるような工夫

表26 は，学校質問紙の質問 (48) の回答に対する各領域の正答率の平均を集計したものである.すべての領域において，肯定的に回答している方が，正答率が高くなっている.

**表26.** 学校質問紙の質問 (48) の回答と各領域の正答率の平均.

	回答		
	1	2	3
数と式	58.4%	56.7%	54.7%
図形	44.6%	43.0%	41.2%
関数	44.3%	42.8%	40.9%
データの活用	57.7%	57.0%	55.8%

表27 は，上記の結果について，分散分析を行った結果である.すべての領域において，5%の有意水準で有意差が認められる.

**表27.** 学校質問紙の質問 (48) の回答と各領域の正答率の平均の分散分析.

	平方和	自由度	平均平方	F 値	有意確率
数と式	77950.781	2	38975.391	34.898	<.001 ^†^
図形	66297.232	2	33148.616	38.878	<.001 ^†^
関数	65240.319	2	32620.160	36.302	<.001 ^†^
データの活用	16975.371	2	8487.6869	9.583	<.001 ^†^

^†^ *p* < .05.

(iii)　評価の観点ごとの正答率のアラインメント

A 実生活における事象との関連を図った授業

表28 は，学校質問紙の質問 (46) の回答に対する知識・技能の観点と思考・判断・表現の観点の平均を集計したものである. 2 つの観点とも，肯定的に回答している方が，正答率が高くなっている.

**表28.** 学校質問紙の質問 (46) の回答と評価の観点別の正答率の平均.

	回答			
	1	2	3	4
知識・技能	60.9%	59.8%	59.0%	55.7%
思考・判断・表現	37.7%	36.3%	34.9%	33.5%

表29 は，上記の結果について，分散分析を行った結果である.両方の評価の観点において，5% の有意水準で有意差が認められる.

**表29.** 学校質問紙の質問 (46) の回答と評価の観点別の正答率の平均の分散分析.

	平方和	自由度	平均平方	F 値	有意確率
知識・技能	31206.399	3	10402.133	13.833	<.001 ^†^
思考・判断・表現	60449.647	3	20149.882	24.021	<.001 ^†^

^†^ *p* < .05.

B 観察や操作，実験等の活動を通して，数量や図形等の性質を見いだす活動

表30 は，学校質問紙の質問(47)の回答に対する知識・技能の観点と思考・判断・表現の観点の平均を集計したものである. 知識・技能の評価の観点では，肯定的に回答している方が，正答率が高くなっている.これに対して，思考・判断・表現の評価の観点においては，1 に回答した正答率の平均が最も高く，その次に 2，4，3 の順になっている.

**表30.** 学校質問紙の質問 (47) の回答と評価の観点別の正答率の平均.

	回答			
	1	2	3	4
知識・技能	61.2%	59.8%	58.9%	58.5%
思考・判断・表現	38.1%	36.2%	35.3%	35.5%

表31 は，知識・技能の結果について，分散分析を行った結果である. 5% の有意水準で有意差が認められる.

**表31.** 学校質問紙の質問 (47) の回答と評価の観点別の正答率の平均の分散分析.

	平方和	自由度	平均平方	F 値	有意確率
知識・技能	45044.777	3	15014.926	19.972	<.001 ^†^

^†^ *p* < .05.

C　公式や決まりなどを指導するとき，生徒がその根拠を理解できるような工夫

表32 は，学校質問紙の質問 (48) の回答に対する知識・技能の観点と思考・判断・表現の観点の平均を集計したものである.2つの観点とも，肯定的に回答している方が，正答率が高くなっている.

**表32.** 学校質問紙の質問 (48) の回答と評価の観点別の正答率の平均.

	回答		
	1	2	3
知識・技能	60.7%	59.2%	57.5%
思考・判断・表現	37.2%	35.7%	33.9%

表33 は，上記の結果について，分散分析を行った結果である. 両方の評価の観点において，5% の有意水準で有意差が認められる.

**表33.** 学校質問紙の質問 (48) の回答と評価の観点別の正答率の平均の分散分析.

	平方和	自由度	平均平方	F 値	有意確率
知識・技能	54316.078	2	27158.039	36.129	<.001 ^†^
思考・判断・表現	60150.190	2	30075.095	35.854	<.001 ^†^

^†^ *p* < .05.

## 4. 達成されたカリキュラムのアラインメントに関する考察

　学校質問紙への回答から把握される授業や指導の実施の程度の回答群間における，算数および数学の正答率の平均の差から，達成されたカリキュラムのアラインメントについて考察する.肯定的な回答，すなわち実施の程度が高くなると，正答率が高くなっている場合には，実施されたカリキュラムである授業や指導が，達成されたカリキュラムである正答率と整合しており，整合的なアラインメントと捉える. このアラインメント分析から，実施された授業や指導と，児童の算数の達成度や生徒の数学の達成度との間に整合的な関連が示唆される. 反対に，整合的なアラインメントが認められない場合は，その授業や指導と算数や数学の学習との間に整合的な関連を示す結果は得られなかったことになる. ただし，本研究は交絡要因（学校規模，地域特性，児童生徒の家庭背景等）を統制した分析ではないため，ここで示される関連は，特定の指導方法が達成度を直接的に高めるという因果的・機能的な関係を主張するものではない.

### (1) 小学校算数

①　実生活における事象との関連を図った授業

　小学校の質問 (57) は，よく実施しているほど，正答率が高くなる整合的なアラインメントが認められた.この事から，実生活における事象との関連を図った授業は，達成されたカリキュラムと機能的に整合していると考えられる.

　しかし，領域ごとのアラインメントを分析すると，数と計算，図形，測定の3つの領域は整合的なアラインメントであったが，変化と関係やデータの活用での2つの領域に関しては，整合的なアラインメントを示す結果は得られなかった.

　また，評価の観点ごとのアラインメント分析では，知識・技能は整合的なアラインメントであったが，思考・判断・表現に関しては，整合的なアラインメントを示す結果は得られなかった.

　これらは，学習指導要領の指導内容である数学的活動に示されている実生活における事象との関連は，実際の授業で実施されていても，児童の身に付けた算数の学習内容とのアラインメントを示す結果は限定的にしか得られなかった.

② 具体的な物を操作するなどの体験を伴う学習を通して，数量や図形について実感を伴った理解をする活動

　質問 (58) は，よく実施しているほど，正答率が高くなるような整合的なアラインメントとはなっていなかった.この事から，具体的な物を操作するなどの体験を伴う学習を通して，数量や図形について実感を伴った理解をする活動と達成されたカリキュラムには、機能的な整合を示す結果は得られなかった.

　領域ごとのアラインメントを分析すると，数と計算に関しては，整合的なアラインメントが認められる.しかし，他の領域や評価の観点ごとの分析では，整合的なアラインメントを示す結果は得られなかった.これより，具体的な物を操作するなどの体験を通した実感を伴った理解をする数学的な活動は，実際の授業で実施されていても，児童が身に付けた算数の学習内容とのアラインメントを示す結果は限定的にしか得られなかった.

③ 公式やきまり，計算の仕方等を指導するとき，児童がそのわけを理解できるような工夫

　質問 (59) は，よく実施しているほど，正答率が高くなるような整合的なアラインメントとはなっていなかった.領域ごとの分析や評価の観点ごとの分析においても，整合的なアラインメントを示す結果は得られなかった.

④ 問題の解き方や考え方の過程が分かるように工夫してノートを書く指導

　質問 (60) は，よく実施しているほど，正答率が高くなる整合的なアラインメントが認められた.領域ごとの分析や評価の観点ごとの分析においても，すべてにおいて整合的なアラインメントが認められた. この事から，問題の解き方や考え方の過程が分かるように工夫してノートを書く指導は，達成されたカリキュラムと機能的に整合していると考えられる.

### (2) 中学校数学

① 実生活における事象との関連を図った授業

　質問 (46) は，よく実施しているほど，正答率が高くなる整合的なアラインメントが認められた. この事から，実生活における事象との関連を図った授業は，達成されたカリキュラムと機能的に整合していると考えられる.

　また，すべての領域の正答率に対して整合的なアラインメントが認められ，評価の観点においても，知識・技能，思考・判断・表現の両方に対して整合的なアラインメントが認められた.数学的活動に含まれている実生活における事象との関連を図った授業と達成されたカリキュラムは機能的に整合していると考えられる.

② 観察や操作，実験等の活動を通して，数量や図形等の性質を見いだす活動

　質問 (47) は，よく実施しているほど，正答率が高くなる整合的なアラインメントが認められた. この事から，観察や操作，実験等の活動を通して，数量や図形等の性質を見いだす活動は，達成されたカリキュラムと機能的に整合していると考えられる.

　ただし，領域ごとの正答率をみると，必ずしも整合的なアラインメントを示す結果は得られなかった領域がある. 数と式とデータの活用の 2 つの領域は整合的なアラインメントが認められたが，図形と関数の 2 つの領域は整合的なアラインメントを示す結果は得られなかった. また，評価の観点ごとの正答率についても，知識・技能の観点には整合的なアラインメントが認められたが，思考・判断・表現の観点には整合的なアラインメントを示す結果は得られなかった.この事から，観察や操作，実験等の活動を通して，数量や図形等の性質を見いだす活動と達成されたカリキュラムとの機能的な整合を示す結果は限定的であった.

③ 公式や決まりなどを指導するとき，生徒がその根拠を理解できるような工夫

　質問 (48) は，よく実施しているほど，正答率が高くなる整合的なアラインメントが認められた. この事から，公式や決まりなどを指導するとき，生徒がその根拠を理解できるような工夫は，達成されたカリキュラムと機能的に整合していると考えられる. 実施の程度も，質問 (46) (47) に比べて，1，2 を回答している割合が高く，本稿で用いた匿名データにおいては 4 を回答している学校は無かった. 公式や決まりなどを指導するとき，生徒がその根拠を理解できるような工夫は，実施されている程度が高い状況にある.

　また，すべての領域の正答率に対して整合的なアラインメントが認められ，評価の観点においても，知識・技能，思考・判断・表現の両方に対して整合的なアラインメントが認められた.公式や決まりなどを指導するとき，生徒がその根拠を理解できるような工夫は，達成されたカリキュラムと機能的に整合していると考えられる.

## 5. おわりに

　本稿では，全国学力・学習状況調査の結果から，中学校数学における達成されたカリキュラムのアラインメントを分析した.

### (1) 研究の結論

達成されたカリキュラムとの間に，整合的なアラインメントの傾向が認められた授業や指導は，次のようにまとめられる.なお，以下に示す関連は，本研究で用いた分散分析の枠組みで観察された群間差を指すものであり，因果関係を主張するものではない.

小学校算数においては，問題の解き方や考え方の過程が分かるように工夫してノートを書く指導を挙げることができる. 数学的な活動である実生活における事象との関連と，具体的な物の操作を通した実感を伴った理解をする活動も，整合的なアラインメントを示す結果は限定的であった. これに対して，公式やきまり，計算の仕方のわけを理解するなどの数学的な活動は，整合的なアラインメントを示す結果は得られなかった.

　中学校数学においては，実生活における事象との関連を図った授業，観察や操作，実験等の活動を通して，数量や図形等の性質を見いだす活動，公式や決まりなどを指導するとき，生徒がその根拠を理解できるような工夫を挙げることができる.ただし，観察や操作，実験等の活動を通して，数量や図形等の性質を見いだす活動のアラインメントの整合性を示す結果は限定的であった. 具体的には，図形と関数の 2 つの領域や思考・判断・表現の評価の観点に対しては，整合的なアラインメントを示す結果は得られなかった.

### (2) 本研究の限界と今後の課題

　本稿では，小学校算数の令和3年度と中学校数学の令和4年度の調査結果の分析を行ったが，現在までの調査結果でも同様の分析が可能であり，同様の結果が得られるのか検証したり、経年変化を分析したりする事が望まれる.また，学校質問紙の質問項目は，どの指導内容がどのように指導されたのか，という詳細について捉えるものではなく，数学的活動も網羅的に取り上げられない事が，こうした大規模調査による全体傾向の把握の限界として指摘できる. こうした意味で，達成されたカリキュラムのアラインメントの概要や一側面を考察したものである. この点に留意した上で，本稿の結果は位置づけられなければならない.

また，本研究では学校単位で得られた学校質問紙の回答を，個票データの各児童生徒に割り当てて分析している. 同一校に所属する児童生徒は同じ説明変数値を共有するため，本分析における観察値は独立ではなく，一元配置分散分析の独立性の仮定を厳密には満たしていない. したがって，本稿で示した検定結果は，全国的傾向の探索的把握として位置づけられるものであり，標準誤差や有意確率は同一校内の児童 生徒の非独立性を考慮して再評価する必要がある. 今後の課題として，学校単位での集計に基づく分析や，児童生徒を学校にネストしたマルチレベル分析等による検証が望まれる.

## Data Availability

全国学力・学習状況調査の集計結果データは，行政機関の保有する情報の公開に関する法律（平成 11 年法律第 42 号）第 5 条第 6 号の規定により保護されており，公表できない.データの利用に当たっては， 「 「全国学力・学習状況調査」の集計結果データの貸与に関わるガイドライン」に基づき，申請を行う事によって貸与が認められ，利用が可能である. ( https://www.mext.go.jp/a_menu/shotou/gakuryoku-chousa/sonota/1386492.htm) （2025.11.23 参照）

## Footnotes

^1^
全国・学力学習状況調査は，義務教育の機会均等とその水準の維持向上の観点から，全国的な児童生徒の学力や学習状況を把握・分析し，教育施策の成果と課題を検証し，その改善を図るとともに，学校における児童生徒への学習指導の充実や学習状況の改善等に役立てる.さらに，そのような取組を通じて，教育に関する継続的な検証改善サイクルを確立する事を目的とした日本国の教育政策の一環として毎年行われる悉皆調査である.この調査の実施については，地方教育行政の組織および運営に関する法律（昭和 31 年法律第 162 号）第 21 条第 17 号の規定により，教育委員会の職務権限において実施されている.この際，調査の目的，内容，調査結果の取扱い等は，書面や口頭で児童生徒及び保護者に通知されており，この調査結果の取扱いは，行政機関の保有する情報の公開に関する法律（平成 11 年法律第 42 号）第 5 条第 6 号の規定により保護されている.この調査実施要領の下，本研究の実施及び匿名データの貸与は，文部科学省の「 「全国学力・学習状況調査」の個票データ等の貸与に関する有識者会議」の審査を経て承諾されたものであり，本稿は，文部科学省総合教育政策局参事官（調査企画担当）付学力調査室の校閲を受け，公開に当たって問題がない事を確認の上，作成されている. なお，所属機関である筑波大学人間系における研究倫理審査は，ヒトに対する調査の計画に対して，調査方法や参加者の同意，データの管理に関しての審査がされている. 本稿で用いている匿名データの元になる全国学力・学習状況調査は，本稿の著作者が計画，実施する調査ではないため，所属機関の研究倫理審査において審査する項目がない. また，仮に審査をしたとしても，その結果に関わらず調査は実施されるため，所属機関の審査対象外である. 審査対象外の調査に対して研究倫理審査を免除する審査は，所属機関では実施されていないため，この調査の実施に対して，本稿の著作者に対する個別の研究倫理審査は不要とされている.

^2^

宮崎 (2019) は，Implemented Curriculum を「実行されたカリキュラム」と日本語訳している.これは「実施されたカリキュラム」と同義であることから，本稿では「実施されたカリキュラム」に統一して表記する.

^3^
1. よく行った，2. どちらかといえば，よく行った，3. あまり行わなかった，4. 全く行わなかった，の 4 つの回答について，数字が小さい方を肯定的と表記する. すなわち，1 > 2 > 3 > 4 の順で肯定的な回答と捉える.
