# Supplementary material for: Attained Curriculum in Japanese Elementary School Arithmetic and Junior High School Mathematics: An Alignment Analysis of the National Assessment of Academic Ability
Source: F1000Res. 2026 Jul 9;15:460. [Article in Japanese] Originally published 2026 Apr 1. [Version 2] doi: 10.12688/f1000research.175939.2 (PMC13644845; doi:10.12688/f1000research.175939.2)
Supplement: Peer review history

# Reviewer report: Reviewer response for version 2

**DOI:** 10.5256/f1000research.203820.r499972

**Published:** 2026-09-09

**Authors:** Toshihito Sato

**Affiliations:** 1 Iwate University, Morioka, Iwate, Japan

査読報告

 本論文の改訂版を確認しました。前回の査読で指摘した事項について、丁寧に検討され、多くの点で適切な修正が行われていました。特に、①「相関関係」という表現を実際の分析内容に即して「回答群間の平均正答率の差を一元配置分散分析により比較する」と修正したこと、②表27の自由度を訂正したこと、③サンプル数と集計表の度数との差について欠測・無回答の扱い及び有効回答率を明記したこと、④参考文献の誤記を訂正したことについては、前回の指摘に適切に対応されています。⑤について、学校質問紙の回答と児童生徒個人のデータとの対応に伴う、同一学校内の児童生徒の非独立性についてです。今回の改訂では、同一校に所属する児童生徒が同じ説明変数値を共有しているため、「一元配置分散分析の独立性の仮定を厳密には満たしていない」ことが明記されました。また、検定結果を「全国的傾向の探索的把握」と位置付け、今後、学校単位での分析やマルチレベル分析による検証が必要であることも記されています。 この追記によって本研究の方法論的限界は明確になりました。

 改訂によって、分析方法及び結果の記述はより明確になったと考えます。

 一方で、以下について、さらに検討するとよいと思いました。

 因果的・機能的な意味を含む表現についてです。今回の改訂版では、考察の冒頭に、本研究で示される関連は「特定の指導方法が達成度を直接的に高めるという因果的・機能的な関係を主張するものではない」と明記されており、前回の指摘を踏まえた重要な修正です。しかし、その後の考察には、「達成されたカリキュラムと機能的に整合していると考えられる」という表現が複数箇所（p20〜21）にあります。例えば、小学校算数の実生活との関連を図った授業やノート指導、中学校数学の実生活との関連を図った授業、観察・操作・実験等の活動、公式や決まりの根拠を理解するための工夫について、この表現が用いられています。 これは、考察冒頭で明記された「因果的・機能的な関係を主張するものではない」という説明と必ずしも整合していないのではないかと思いました。「達成されたカリキュラムとの間に整合的な関連が示唆された」「整合的な傾向が認められた」等、本研究の分析から直接支持できる範囲の表現に統一することが適切と考えます。

 軽微な点ですが、「5．おわりに」の冒頭が「中学校数学における達成されたカリキュラムのアラインメントを分析した」となっています。本研究は小学校算数も対象としているため、「小学校算数および中学校数学における」とするのがよいと思われます。

 前回の指摘の多くについては適切な修正がなされており、よい論文であると思いました。一方で、「機能的に整合している」という表現については、もう少し検討してもよいかと考えます。

（該当する場合は要回答）統計分析および解釈は適切ですか。

一部該当

本研究は明確かつ正確に提示されたものであり、最新の文献を引用していますか。

はい

方法と分析について第三者による再現が可能となるよう十分な詳細が提示されていますか。

一部該当

結果の基礎となるソースデータはすべて入手可能で再現性を十全に保証していますか。

はい

結論は結果により妥当な裏付けを得ていますか。

一部該当

研究設計は適切で学術的価値がありますか。

はい

Reviewer Expertise:

数学教育

I confirm that I have read this submission and believe that I have an appropriate level of expertise to confirm that it is of an acceptable scientific standard.

# Reviewer report: Reviewer response for version 1

**DOI:** 10.5256/f1000research.193962.r484987

**Published:** 2026-06-03

**Authors:** Yasufumi Kuroda

**Affiliations:** 1 Kyoto University of Education, Kyoto, 612-0863, Japan

本論文では、小学校算数および中学校数学における達成されたカリキュラム（ここで言う達成カリキュラムとは、児童・生徒に獲得することが期待される学習内容が，実際に児童・生徒に獲得されたのかというもの）に焦点化し、全国学力・学習状況調査の結果を対象に、教育におけるアラインメントの分析を行っています。なお、ここでいう教育のアラインメントとは、「教育における目標・指導・評価が整合的かつ機能的に整列している状態」としています。

 本研究は、これまでの先行研究における「意図されたカリキュラム」と「実施されたカリキュラム」のアラインメントを取り上げたものなどとは異なり、「達成されたカリキュラム」を研究対象として、全国学力・学習状況調査の結果をもとにアラインメントを分析している点にオリジナリティがあるといえます。

 　対象としたデータは小学校算数においては、全国の小学校第6学年の約10％にあたる99,455人であり、統計的考察において十分な数を確保しているといえます。また、抽出した児童と全体の成績結果との比較においては、大きな差異は見られず、全体を代表するデータとなっています。

 中学校数学においては、全国の小学校第3学年の約10％にあたる89,435人であり、こちらも統計的考察において十分な数を確保しているといえます。また、抽出した児童と全体の成績結果との比較においては、大きな差異は見られず、全体を代表するデータとなっています。

 両者とも、各項目に関する数値の整理と、基本的な統計的分析がなされています。

 「4. 達成されたカリキュラムのアラインメントに関する考察」において、小学校算数における整合的なアラインメントを示す結果とならなかった事項として、「変化と関係やデータの活用での2つの領域」、「評価の観点における思考・判断・表現」を指摘しています。具体的には、通常の算数科で重視されている「具体的な物を操作するなどの体験を伴う学習を通して，数量や図形について実感を伴った理解をする活動」が、達成されたカリキュラムへの整合性を持つものとは言えないといった指摘がなされており、今後の研究へとつながる重要な成果が見られます。一方で「問題の解き方や考え方の過程が分かるように工夫してノートを書く指導」において、達成されたカリキュラムへの整合性が高いという本論文では、小学校算数および中学校数学における達成されたカリキュラム（ここで言う達成カリキュラムとは、児童・生徒に獲得することが期待される学習内容が，実際に児童・生徒に獲得されたのかというもの）に焦点化し、全国学力・学習状況調査の結果を対象に、教育におけるアラインメントの分析を行っています。なお、ここでいう教育のアラインメントとは、「教育における目標・指導・評価が整合的かつ機能的に整列している状態」としています。

 本研究は、これまでの先行研究における「意図されたカリキュラム」と「実施されたカリキュラム」のアラインメントを取り上げたものなどとは異なり、「達成されたカリキュラム」を研究対象として、全国学力・学習状況調査の結果をもとにアラインメントを分析している点にオリジナリティがあるといえます。

 　対象としたデータは小学校算数においては、全国の小学校第6学年の約10％にあたる99,455人であり、統計的考察において十分な数を確保しているといえます。また、抽出した児童と全体の成績結果との比較においては、大きな差異は見られず、全体を代表するデータとなっています。

 中学校数学においては、全国の小学校第3学年の約10％にあたる89,435人であり、こちらも統計的考察において十分な数を確保しているといえます。また、抽出した児童と全体の成績結果との比較においては、大きな差異は見られず、全体を代表するデータとなっています。

 両者とも、各項目に関する数値の整理と、基本的な統計的分析がなされています。

 　「4. 達成されたカリキュラムのアラインメントに関する考察」において、小学校算数における整合的なアラインメントを示す結果とならなかった事項として、「変化と関係やデータの活用での2つの領域」、「評価の観点における思考・判断・表現」を指摘しています。具体的には、通常の算数科で重視されている「具体的な物を操作するなどの体験を伴う学習を通して，数量や図形について実感を伴った理解をする活動」が、達成されたカリキュラムへの整合性を持つものとは言えないといった指摘がなされており、今後の研究へとつながる重要な成果が見られます。一方で「問題の解き方や考え方の過程が分かるように工夫してノートを書く指導」において、達成されたカリキュラムへの整合性が高いという指摘は、ICT機器の活用とノートの活用といった点のバランスを考察する上での重要な研究成果であるといえます。

 中学校数学における整合的なアラインメントを示す結果とならなかった事項として、「図形と関数の2つの領域」、「評価の観点における思考・判断・表現」を指摘しています。領域は小学校と異なりますが、評価の観点は小学校と同一であることから、この点が日本の算数・数学の教育的課題であることを示しています。また、「実生活における事象との関連を図った授業、観察や操作、実験等の活動を通して、数量や図形等の性質を見いだす活動」において、達成されたカリキュラムへの整合性が高いという指摘は中学校数学の授業のあり方を考えるうえでの重要な示唆を与えています。

 「本研究の限界」においても記されていますが、継続的な研究による経年変化分析（とりわけ学習指導要領改訂前後）や、調査対象数を減じた中での詳細な質問項目に関する調査と分析などを組み合わせることで、より精緻な研究になることが期待されます。

（該当する場合は要回答）統計分析および解釈は適切ですか。

はい

本研究は明確かつ正確に提示されたものであり、最新の文献を引用していますか。

はい

方法と分析について第三者による再現が可能となるよう十分な詳細が提示されていますか。

はい

結果の基礎となるソースデータはすべて入手可能で再現性を十全に保証していますか。

はい

結論は結果により妥当な裏付けを得ていますか。

はい

研究設計は適切で学術的価値がありますか。

はい

Reviewer Expertise:

数学教育学、教育工学

I confirm that I have read this submission and believe that I have an appropriate level of expertise to confirm that it is of an acceptable scientific standard.

# Response

**Published:** 2026-06-12

**Authors:** Naomichi Makinae

**Affiliations:** Institute of Human Sciences, University of Tsukuba, Tsukuba, Ibaraki, Japan

I kindly ask you to carefully read my article, "Achieved Curriculum in Elementary and Junior High School Mathematics in Japan: An Alignment Analysis of the National Academic Achievement and Learning Status Survey,"

 Thank you for your reviewer comments. I appreciate that you clearly understood and evaluated the arguments in this paper. Another reviewer raised concerns about the rigor of this manuscript, and I am submitting a revised version correcting those points. Thank you for your understanding.

# Reviewer report: Reviewer response for version 1

**DOI:** 10.5256/f1000research.193962.r474504

**Published:** 2026-05-04

**Authors:** Toshihito Sato

**Affiliations:** 1 Iwate University, Morioka, Iwate, Japan

修正・検討案

 ①　「相関関係を求めた」など相関関係という記述が複数箇所に見られますが，実際に行われているのは４件法の複数の質問紙調査項目を群ごとに分け算出した平均値及びその比較，分散分析です。「相関関係を求めた」というよりは「回答群間の平均正答率の差を比較した」や「回答群間の平均正答率の差を分散分析により比較した」のように，実際の分析内容に即した記述にしてはどうでしょうか。

 （該当箇所　p.1, l.17–20　p.8, l.3–12　p.15, l.6–15　p.9, l.2–3　p.16, l.7–40）

 ②　表27について。質問（48）では表20，26で回答群が3つになっていますので，表27においては自由度が「2」になるのではないでしょうか。よって，表27の平方平均の計算についても再計算が必要になります。

 ③　小学校サンプルは99,445人と書かれていますが，表3の度数合計は99,159で，286人分の差があります。中学校も89,435人に対し表20の各項目合計は89,170で，265人分の差があります。欠測・無回答・除外の扱いを説明する必要はありませんか。また項目ごとの有効回答率も若干も変わるのでしょうか。

 ④　参考文献の清水ら(2021)は，本文p.3, l.19–20では「中学校数学」と紹介されていますが，最後の参考文献では抽出上「中学校算数」となっています。「中学校数学」に訂正をしてください。

 ⑤　学校質問紙は学校が回答するものだと本文で説明されています。一方で，集計表の度数は小学校で約9.9万人，中学校で約8.9万人規模であり，学校数ではなく児童生徒数に見えますので，何か断りをしてもよいでのはないでしょうか。学校のレベルを説明変数とし，児童生徒の成績レベルに対応させていくためには，同一学校内の生徒の非独立性を考慮する必要はないでしょうか。

 ⑥　本稿が実際に示しているのは，学校質問紙の回答傾向と正答率との関連であり，考察・結論では，特定の指導が「整合的なアラインメント」として機能している，あるいは学習目標の実現を支えると読める表現があります（要旨p.1-2，考察・結論部分）。現状の分析では交絡要因の統制が十分ではなく，因果的あるいは機能的な意味合いまで述べるのは強すぎるように思われます。「関連がみられた」「整合的な傾向が示唆された」程度にとどめるのが適切ではないでしょうか。

（該当する場合は要回答）統計分析および解釈は適切ですか。

一部該当

本研究は明確かつ正確に提示されたものであり、最新の文献を引用していますか。

はい

方法と分析について第三者による再現が可能となるよう十分な詳細が提示されていますか。

一部該当

結果の基礎となるソースデータはすべて入手可能で再現性を十全に保証していますか。

はい

結論は結果により妥当な裏付けを得ていますか。

一部該当

研究設計は適切で学術的価値がありますか。

はい

Reviewer Expertise:

数学教育　学力アセスメント

I confirm that I have read this submission and believe that I have an appropriate level of expertise to confirm that it is of an acceptable scientific standard, however I have significant reservations, as outlined above.

# Response

**Published:** 2026-06-12

**Authors:** Naomichi Makinae

**Affiliations:** Institute of Human Sciences, University of Tsukuba, Tsukuba, Ibaraki, Japan

Explanation

**of Corrections:** Please read my paper "Achieved Curriculum in Japanese Elementary and Junior High School Mathematics - Alignment Analysis of the National Academic Achievement and Learning Status Survey," Thank you for your important feedback. By reviewing your feedback, we gained many insights to improve the quality of this article. We have revised the text, tables, and references, and below are explanations in response to your feedback. The corrections are recorded in Word proofreading under "Change History Logs."

**Observation (1) Regarding the description of 'correlation relationships'**

 There are multiple references to correlation in various places, such as 'correlation was found,' but in practice, the analysis is calculated by dividing multiple questionnaire survey items from the four methods into groups, including the average, comparison, and analysis of variance. Rather than "finding a correlation," how about using descriptions that reflect the actual analysis content, such as "comparing the difference in average correct response rates between response groups" or "comparing differences in mean correct response rates between response groups using ANOVA"?

 (Relevant passages: p.1, l.17–20, p.8, l.3–12, p.15, l.6–15, p.9, l.2–3, p.16, l.7–40)

 As you pointed out, what was conducted in this study was not the derivation of correlation coefficients, but rather a comparison of average correct response rates between response groups (one-way ANOVA), so the use of the term 'correlation' was inappropriate. The points you pointed out (p.1, l.17–20, p.8, l.3–12, p.15, l.6–15, p.9, l.2–3, p.16, l.7–40) have been standardized and corrected as follows.

 "Investigating whether there is a correlation," → "Examining whether there is a difference in mean correct answer rates among response groups using one-way ANOVA (significance level 5%),"

 "Considering from correlations..." → "Considering differences in mean correct response rates among response groups"

**Observation (2) Regarding degrees of freedom in Table 27** Regarding

 Table 27. In question (48), since Tables 20 and 26 show three groups, wouldn't the degree of freedom in Table 27 be '2'? Therefore, the square mean calculation in Table 27 also needs to be recalculated.

 As you pointed out, the degrees of freedom in Table 27 are typographical errors, and the correct number is '2.' This also matches the fact that question (48) in Table 21 lists degrees of freedom as '2.' Note that the value for the mean square is calculated with degrees of freedom 2, so this is an error in the notation of degrees of freedom. There was no need to recalculate the mean square. In response to your feedback, we revised the degrees of freedom in Table 27 to '2' and double-checked the entire table.

**Observation (3): Differences between sample size and total frequency, and handling**

 of missing data The elementary school sample is listed as 99,445 people, but the total prescription in Table 3 is 99,159, a difference of 286 people. For junior high schools, there are 89,435 students, compared to the total of 89,170 for each item in Table 20, a difference of 265 students. Is it necessary to explain how to handle missing tests, no responses, or exclusions? Also, will the effective response rates vary slightly by item?

 As you pointed out, the explanation was insufficient. The difference in total frequency is due to non-responses or missing entries for each item on the school questionnaire. Main text (Elementary School Mathematics 3. (1)(1)(i) and Junior High School Mathematics 3. The following content has been added to (2)(1)(i)).

 ・Elementary schools: Number of valid responses per question (57 questions) 99,159 people; Questioning (58) 99,253 people; Questions (59) 99,209 people; Question (60) 99,197 people, all with valid response rates of 99.7–99.8%) were clearly stated.

 ・Junior High School: It was clearly stated that 89,170 students who received valid answers on all three questions (valid response rate of 99.7%) were included in the analysis.

 In each analysis, we also described that the list wise exclusion was adopted only for cases where valid responses were provided to the question item, and that since the valid response rate exceeded 99.7% for both cases, the bias caused by missing tests is considered limited.

**Point-of-note (4) Regarding the bibliographic information of Shimizu et al. (2021**) in the reference list, Shimizu et al. (2021) is introduced as 'junior high school mathematics' on pages 3 and l.19–20 of the main text, but in the final reference, it is extracted as 'junior high school mathematics.'

 Please correct it to 'Junior High School Mathematics.'

 As you pointed out, the literature information was incorrect. In the reference list, Shimizu et al. (2021) changed the subtitle from "Focusing on the Junior High School Mathematics Domain 'Functions'" to "Focusing on the Junior High School Mathematics Domain 'Functions'" I have revised it to the official title of the original text. After checking the original text, I have revised the subtitles for the research conference session and the following Shimizu et al. (2022) to 'Arithmetic' → 'Mathematics Department.'

**Pointout (5): Units**

 of Analysis and Non-Independence The school questionnaire is explained in the text that the school answers the questions. On the other hand, the frequency in the summary table is about 99,000 in elementary schools and about 89,000 in junior high schools, so it appears to be the number of students rather than schools, so perhaps it is appropriate to make some objections. To use school level as an explanatory variable and match the academic performance levels of students to the same school, isn't it necessary to consider the independence of students within the same school?

 This is an extremely important point and has been carefully considered as it concerns the methodological limitations of this research.

 First, as an explanation of the analysis units, we have added the following explanation at the beginning of the sections on elementary and junior high school mathematics.

 ・The anonymous data used in this study are provided on a per-sheet basis, and each student's record is assigned responses to the school's school questionnaire.

 ・Therefore, the frequency in the summary table must represent the number of students and students.

 Second, regarding the inindependence caused by students belonging to the same school sharing answers to the same school questionnaire, as stated in "5. (2) Limitations of this research and future challenges," with a new paragraph added, clearly stating the following contents.

 ・The observations in this analysis are not strictly independent and do not strictly satisfy the assumption of independence in one-way ANOVA.

 ・The test results presented in this paper should be positioned as an exploratory grasp of national trends.

 ・As a future task, it is desirable to conduct analysis based on school-level aggregation and verification through multilevel analysis that nests students at schools.

 This study positions the alignment of the achieved curriculum as a pioneering attempt to analyze anonymous data from the National Academic Achievement and Learning Status Survey. While this study focuses on exploratory understanding, we have clarified its limitations based on your feedback.

**Pointout (5) Regarding the expression of causal and functional interpretation**, this paper actually shows the relationship between response trends and correct response rates in school questionnaires, and in consideration and conclusion, specific instruction functions as a 'consistent alignment.'

 Alternatively, there are expressions that can be read as supporting the achievement of learning objectives (Summary p.1-2, Discussion & Conclusion Section)Points). In the current analysis, controlling confounding factors is insufficient, and it seems too strong to discuss causal or functional implications. Wouldn't it be appropriate to limit it to something like "a correlation was observed" or "a consistent trend was suggested"?

 As you pointed out, we have completely revised the expression.

 First, at the beginning of Consideration (4), we redefined "consistent alignment," clarifying that this refers to the observed relationship as the average difference in correct response rates between response groups, and does not claim causation.

 Second, in the text of Discussion and Conclusion (5), the following terms have been replaced.

 ・"Considered functionally consistent→ "Suggests an alignment association," "Observed a consistent association,"

 "Functionally consistent with the achieved curriculum," → "Consistent association with the achieved curriculum,"

 "Consistent alignment observed," → A tendency toward consistent alignment was observed.

 Thirdly, 5. In section (2), it is clearly stated that this analysis does not control for confounding factors (such as school size, regional characteristics, or the family background of students and students), and the observed associations do not indicate causation.

 Similarly, for the abstract, expressions with causal implications have been replaced with phrases such as "a correlation was observed" or "a trend was suggested."

 From these revisions, we have improved the accuracy of the thesis's descriptions ((1)(2)(4)), data accuracy ((3)), clarification of methodological limitations ((5)), and reasonable strength of conclusions ((6)). All of these points are important and relevant to the core of this research, and we deeply appreciate them.

 We would appreciate it if you could check it out. Thank you for your continued support.
